# Social and Behavioral Correlates of Adolescent Sexual Experience and Intention to Use Condoms in Northwestern Botswana

**DOI:** 10.3390/ijerph18115583

**Published:** 2021-05-24

**Authors:** Francis Barchi, Helen Apps, Oleosi Ntshebe, Peggie Ramaphane

**Affiliations:** 1 Edward J. Bloustein School of Planning & Public Policy, Rutgers-The State University of New Jersey, New Brusnwick, NJ 08901, USA; 2 WoMen Against Rape, Maun, Botswana; helenapps@womenagainstrape.org.bw (H.A.); peggie.ramaphane@womenagainstrape.org.bw (P.R.); 3 Department of Population Studies, University of Botswana, Gaborone, Botswana; ntshebe@ub.ac.bw

**Keywords:** adolescents, HIV-risk behavior, gender norms, condoms, sexual debut, Africa

## Abstract

Adolescent sexual behavior is shaped by individual, social, and structural factors that can increase HIV-risk, unwanted pregnancy, and sexually transmitted disease. To inform the development of a comprehensive sexuality education program, 239 secondary school adolescents ages 14–19 in Maun, Botswana, completed a survey of sexual and reproductive health knowledge, attitudes, and behaviors in February–March 2020. Bivariate and multivariate analyses examined factors associated with sexual experience and perceived ability to insist on condoms. Approximately 21% of respondents reported having had sexual intercourse. More than half felt able to insist on condoms. Sources of information about human reproduction, alcohol use, attitudes about when sex is acceptable, and perceived sexual activity by one’s peers were predictive of sexual experience. Age, confidence in correct condom use, perceived acceptability of adolescent sex with condoms, and endorsement of prevailing gender norms were significantly associated with perceived ability to insist on condom use.

## 1. Introduction

Eighty-eight percent of all new adolescent HIV infections occur in Sub-Saharan Africa [1]. Girls disproportionately bear the burden of HIV in the region, accounting for 83% of all new infections in eastern and southern Africa. Adolescents are often limited in their ability to access sexual and reproductive health information, resources, and adolescent-friendly clinical care [2]; girls can be unable to negotiate safe sex or to seek care or post-exposure prophylaxis to prevent HIV transmission, or emergency contraceptives to prevent unwanted pregnancy [3]. Norms that reinforce the subordinate role of girls and increase their vulnerability to HIV, unintended pregnancies, and various forms of abuse also shape how boys view ‘masculine‘ conduct, relationships, and risk behaviors [4,5]. Unprotected sex is a common denominator in both HIV infections and pregnancies in this population. 

A considerable body of scholarly work has used an ecological model as a framework for characterizing the protective and risk factors associated with adolescent sexual behavior, particularly in the context of HIV and other sexual transmitted diseases [6,7,8]. A systematic review of the literature on adolescents in developing countries published between 1990 and 2010 found that most studies focused on one of two outcomes: sexual initiation and condom use [9]. A number of risk factors appear to be consistently predictive of sexual initiation, including, at the level of the individual, being older, male, married, and/or using alcohol, and at the peer level, having friends who have had sex [9,10,11]. Being in school was found to be a protective factor against early sexual initiation in the majority of studies, and condom use was positively associated with more years of education and being able to talk with one’s partner about sex [9]. A study of 33 countries in Sub-Saharan Africa published in 2020 suggested that, despite similarities in risk factors across the region, there are significant and persistent inequalities in adolescent sexual and reproductive health indicators that result from differences in education and urban–rural residence [12]. 

### Adolescent Sexual and Reproductive Health in Botswana

Botswana’s government has committed to improving sexual and reproductive health (SRH) services and educational programming for adolescents. The Ministry of Basic Education has called for the incorporation of comprehensive sexuality education (CSE) curricula at the primary, secondary, and teacher-training levels, and for the establishment of guidance and counselling units in schools. The government has also begun to establish adolescent-friendly sexual and reproductive health (SRH) services in clinics and health centers. Resource constraints have limited progress in both of these areas. 

Despite such efforts and despite major advances in reducing HIV-incidence overall, young people continue to be a highly vulnerable segment of the country’s population, with HIV-prevalence rates among adolescents 15–19 years of roughly 3% for males and 6% for females in 2013 [13]. In 2018, nearly 25% of these new infections occurred in adolescent girls and young women ages 15–24 [14]. Some researchers have attributed this to cultural norms that reinforce male dominance and marginalize women from decision-making [15]. Unprotected sex and lack of knowledge about SRH contribute to high HIV-infection rates and unintended pregnancies in this age cohort. According to a rapid assessment survey undertaken by UNFPA and the Botswana Ministry of Basic Education in 2017, the adolescent fertility rate was 51 births per 1000 [16]. In 2015, 813 young women, roughly 43% of those who left secondary school, attributed their dropping out to pregnancy [17,18]. A 2018 study of adolescents in Gaborone, Botswana’s capital city, found that nearly 37% of male and 21% of female adolescents reported having had their first sexual intercourse by the age of 15 (Std. Dev. 2.4) [19].

Despite a growing body of research examining risk factors for adolescent sexual and reproductive health in Sub-Saharan Africa, including studies specific to Botswana, there has not been any focused examination to date of the risk and protective factors associated with adolescent sexual risk behaviors in the villages and towns that are home to more than 57% of Botswana’s population [20].The current study was undertaken prior to the implementation of a new sexuality education program being implemented in a large village in northwestern Botswana in order to provide a foundational understanding of the knowledge, attitudes, and behaviors of school-going adolescents in this setting and to establish a baseline by which to evaluate the new program’s effectiveness. We hypothesized that sources of SRH information and gendered attitudes about relationships would be significant predictors of adolescent sexual experience and condom use.

## 2. Materials and Methods

This analysis uses cross-sectional data collected in February–March 2020 to examine SRH knowledge, attitudes, and behaviors of school-going adolescents ages 14–19 years in northwestern Botswana. The study is part of a multiyear program under the direction of WoMen Against Rape (WAR), a human rights organization based in Maun, to increase the availability and accessibility of SRH information and resources for adolescents and to contribute to the development of a CSE curriculum for use in the Northwest District secondary schools. The Aka! Re Koo! Project (translated from Setswana, the local language of Botswana, as ‘Hey! We are here!’ and colloquially referred to as the ‘ARK project’), aims to promote adolescent SRH by reducing risk behaviors and addressing norms around gender and intimate relationships that give rise to HIV, STIs, and teen pregnancy in this population. Maun is a large village/town in northwestern Botswana with a population of approximately 56,000; many of the residents of Maun are poor, relying on subsistence farming, incomes from small herds of goats or cattle, and intermittent work in the informal sector. 

### 2.1. Sample

For the purposes of this research, we used secondary school adolescents ages 14–19 as the cohort of interest to fall within the definition of ‘adolescent’ adopted by the World Health Organization, UNICEF, and UNAIDS, i.e., persons ages 10–19 [21]. Study participants were recruited from adolescents who had recently signed up to be part of new ARK Clubs that are being established by WAR in each of the six public secondary schools in Maun. ARK Clubs, once fully operational, will meet weekly on school premises and engage approximately 300 students in activities designed to help adolescents build social and emotional skills, understand sexuality and desire, and be better informed about SRH resources. Each school’s ARK Club will include 25 males and 25 females ages 14–19.

At the time of the study, six secondary schools had agreed to establish ARK Clubs, and 300 students had signed up as members. Principals at each of the participating schools gave permission to have the study administered on the school premises during school hours. Only five ARK Clubs participated in the surveys prior to COVID-19-related school closures went into effect in Botswana, resulting in a maximum sample size of 250 students from five participating school clubs. Two students did not have parental permission to participate, and an additional eight students were not present in their club meetings on the days when surveys were administered. Of the 240 self-administered surveys that were collected, one was blank, and six had missing responses on gender, a critical variable in our study; these were omitted from the analytic sample of 233 respondents.

### 2.2. Measures

The outcome variables of interest in this study were sexual experience, defined as ever having had sexual intercourse, and perceived ability to insist on condom use. To examine these two phenomena, a semi-structured survey instrument was adapted for use from the Illustrative Questionnaire for Interview Surveys with Young People and the 2005 Botswana Global School-Based Student Health Survey (GSHS) [22,23], and pre-tested in a sample of 27 students attending a private secondary school in Maun not participating in the study. The survey included the following domains of questions: demographic information; respondent risk behaviors with respect to smoking, alcohol and substance use, and unprotected sex; sexual relationships and experiences; sources of SRH information; knowledge and attitudes toward condom use; and views on relationships. Five statements were used to capture respondent perceptions on gender norms around intimate relationships: ‘I think sometimes a boy has to force a girl to have sex if he loves her’; ‘it is sometimes justifiable for a boy to hit his girlfriend’; ‘men need sex more frequently that women do’; ‘men have a right to have sex with their girlfriend whenever they wish’; and ‘if a girl has accepted cash or gifts from a man, she must have sex with him if he wants it’. Respondents were asked to indicate whether they agreed, disagreed, or were uncertain as to their views on each statement. The question ‘how many of your friends have had sexual intercourse’ was included to capture respondent perceptions of the sexual experiences of their peers. Survey questions were available in English and Setswana. All education from Standard 4 (equivalent to US 4th grade) takes place in English, the ‘official’ language of the country. All participants in the study were therefore likely to speak both languages with ease but may have had a preference to read and respond in one or the other language. 

### 2.3. Administration of the Surveys

At the start of the ARK Club meetings during which the surveys were administered, a WAR staff member explained the purposes of the study and use of the data, the risks and benefits associated with participation, and the rights of participants. Students received an information sheet about the study that they were free to retain and a paper copy of the survey to complete if they chose to do so. 

### 2.4. Ethical Considerations

This study was developed in accordance with the World Health Organization’s guidance on ethical considerations in planning and reviewing research studies on sexual and reproductive health in adolescents [16]. Although Botswana legally recognizes persons 18 years and older as adults, it remains customary for unmarried children still attending school and residing in their natal homes to remain under the decisional authority of a parent, guardian, or older male relative regardless of age. With the approval of all ethics committees involved in the review of the protocol, this study provided parents and guardians with study information and the opportunity to ‘opt out’ with respect to their child’s participation. Four of the five principals of secondary schools participating in the study adopted this passive consent model and distributed letters to parents notifying them of the intent to survey ARK Club members and requesting that they communicate any concerns or refusals to the school. One principal, however, required documentation of active consent by asking parents to sign and return permission letters. 

All students who had parental permission to participate were provided with information about the study at the start of the ARK Club meetings in which the surveys were to be administered. They were advised that they were not required to participate nor to answer any questions they did not wish to answer. A cover sheet attached to the survey requested that a student tick a box to indicate consent to participate prior to moving on to respond to study questions. No names or personal health information were collected. 

### 2.5. Analysis Strategy

All data were analyzed using Stata statistical software version 16 (StataCorp, LLC, College Station, TX, USA) [24]. Frequencies were run for all variables, and bivariate analyses were conducted to examine their association with sexual intercourse and gender. Missing observations were excluded in the calculation of Pearson’s chi-square and Fisher’s exact test statistics. Logistic regression models containing those attributes shown to have associations that were significant at *p* < 0.05 were run for the two outcome variables of interest. Variables that were significantly associated with gender were included in the model examining ‘able to insist on condom use’. The multilevel categorical variable ‘able to insist on condom use’ was collapsed into a dichotomous yes/no variable for the regression models. Only one of two variables reflecting attitudes about men’s sexual behaviors (‘men need sex more frequently’ and ‘men have a right to sex’) was included in the logistic regression models given the potential problem of collinearity; including the variable ‘men need sex more frequently’ achieved the best model fit and was used for the analysis. List-wise deletion was used to remove those observations that did not have responses to all the variables included in the models. 

A post hoc power analysis using the sample size of 233 was conducted with the statistical software, G*Power [20]. The effect sizes for this analysis were: small (f _2_ = 0.10), medium (f _2_ = 0.30), and large (f _2_ = 0.50) [25], with an alpha level of *p* < 0.05. This analysis indicated that the statistical power was 0.33 for detecting a small effect, and more than 0.99 for detecting medium and large effects. There was more than adequate power (* 0.80) to detect moderate to large effects but inadequate statistical power to detect a small effect. Variance inflation factors were computed using Stata’s VIF command to test for multicollinearity in both models [26]. The mean VIF in each model was 1.72, generally considered within an acceptable range [27].

## 3. Results

Frequencies and the association of attributes with having had sexual intercourse and gender are reported in Table 1 and Table 2, respectively. The majority of respondents were female, fifteen years of age, and lived in households headed by their fathers. Approximately 90% or more did not smoke cigarettes, consume alcohol, or use illegal drugs, although many adolescents reported that they had previously tried cigarettes and/or alcohol. The majority of respondents relied on family members or teachers for SRH information. Approximately 21% of respondents reported having had sexual intercourse; the majority of these had sexual intercourse for the first time at age 15 years and were male. Sexual experience was significantly associated with gender, age, cigarette/alcohol/substance use, and sources of SRH information. Gender was significantly associated with sexual experience, drug use, and information sources. 

The distribution of knowledge and use of condoms and views on relationships according to sexual experience and gender are reported in Table 3 and Table 4, respectively. The majority of respondents agreed with statements about condom efficacy in preventing pregnancy and protecting against HIV and other sexually transmitted diseases; responses did not vary significantly according to gender or sexual experience. While more than 40% of respondents thought that it was all right for boys and girls to have sex as long as they used condoms, less than a third of all respondents felt that they knew how to use a condom correctly. Significantly more females than males agreed with the statement ‘men need sex more frequently than women do’, (χ^2^ (2, *n* = 229) =14.92, *p* = 0.001), while more males than females felt that men had a right to have sex with their girlfriends whenever they wished (χ^2^ (2, *n* = 219) = 16.15, *p* < 0.001). More than half of adolescents felt confident that they could insist on condom use every time they had sex. Confidence in knowing the correct use of condoms and in the ability to insist on condom use varied significantly with both gender and sexual experience.

### Multivariable Analyses

The results of logistic regressions examining the characteristics and behaviors associated with adolescent sexual experience and perceived ability to insist on condom use are reported in Table 5. Controlling for other attributes, believing that it is all right to have sex as long as condoms are used and never having consumed alcohol significantly increased the odds that a respondent would report having had sexual intercourse. Respondents who believed that sex between boys and girls was all right provided condoms were used had greater than four times the odds of having had sexual intercourse compared to those students who disagreed with the statement. Respondents were 84% less likely to report sexual experience if they relied on teachers for information about human reproduction than if that information came from family members. Students who did not consume alcohol had 83% lower odds of reporting sexual experience than students who did. While the model suggested a significant association between respondents’ perceptions of their friends’ sexual behaviors and their own, the extremely large 95% confidence interval suggests that this is an unstable finding and caution should be used in its interpretation.

Respondents’ perceived ability to insist on condom use every time they had sexual intercourse was significantly associated with age, confidence that they knew how to use a condom correctly, and three statements reflecting views on relationships. Fifteen year olds had 2.4 times the odds of believing that they could insist on condom use compared to fourteen year olds when controlling for other factors. Students who disagreed or said they did not know in response to the statement ‘I know how to use a condom correctly’ had 77% and 83% lower odds, respectively, of believing that they could insist on condom use than those students who agreed with the statement. Students who agreed that men needed sex more frequently than women had 4.6 times greater odds of stating that they could insist on condom use than students who did not. Agreeing or being unsure about the statement ‘sometimes a boy must force his girlfriend to have sex if he loves her’ decreased the odds that students would be confident that they could insist on condom use by 68% and 88%, respectively. Similarly, those students who agreed with or were uncertain about the statement ‘it is all right for boys and girls to have sex if they use a condom’ had 62% and 87% lower odds of believing themselves capable of insisting on condom use, respectively, than those who agreed. Sexual experience was not predictive of perceived ability to insist on condoms after controlling for sociodemographic factors, attitudes, and information sources.

## 4. Discussion

This study examines various associations between the characteristics of adolescents in northwestern Botswana, their attitudes toward various sexual and reproductive health topics, and two behaviors: having had sexual intercourse and insistence on condom use. While other studies have looked at adolescent HIV-risk behaviors, sources of information about sex, and perceived social norms around male and female behaviors in Botswana, most recently in 2018, these studies have drawn their samples from Gaborone, Botswana’s capital city and largest urban area. This study is, to the authors’ knowledge, the first attempt to examine adolescent SRH knowledge, attitudes, and behaviors in a more rural area where access to SRH information, health services, and school-based sexuality programs is more limited. It provides baseline data for evaluation of a new sexuality education program in this setting and points to areas of future inquiry on these topics. Importantly, more than one half of adolescents had their first sexual intercourse at age 15, a finding that would suggest that educational intervention to improve adolescent appreciation of sexual risk should occur in this setting prior to that age, or in the first year of secondary school. The associations between three factors and sexual experience were found to be significant when controlling for covariates: never having consumed alcohol, relying on teachers as sources of information about the reproductive system, and the perception that sex between adolescent boys and girls was acceptable as long as condoms were used. Adolescents’ confidence in their ability to insist on condom use in their sexual encounters was significantly associated with perceived knowledge about how to use condoms correctly and agreement with the statement ‘men need sex more frequently than women’. 

Early sexual debut has been identified as a significant risk factor for HIV infection and unintended pregnancy among adolescents in Sub-Saharan Africa [10,28,29]. Data from the current study suggest that there is a significant increase in reported sexual experience that occurs when adolescents reach age 15 years (χ^2^ (2, *n* = 230) = 13.08, *p* = 0.001). This finding is similar to that of an earlier study of urban adolescents in Botswana which suggested that adolescents become sexually active between 15–17 years of age [30] as well as findings from a large multi-country analysis that found that adolescent sexual debut in Sub-Saharan Africa occurred on average between the ages of 15–18 years [29]. 

Although findings at the bivariate level in this analysis identify a number of attributes, such as gender, age, and alcohol use, which are widely identified in the literature as predictive of adolescent sexual experience, only alcohol use (OR 0.17, *p* = 0.029, 95% CI [0.03, 0.83]) and teachers as trusted sources of SRH information were found to be significant when sources of SRH information, perceived peer behavior, and views on relationships were introduced into the multivariable model examining sexual experience. When adolescents identified teachers rather than family members as important sources of SRH information, the odds that they would have engaged in sexual intercourse were significantly reduced (OR 0.16, *p* = 0.010, 95% CI [0.04, 0.65]). This finding aligns with earlier studies that found formal education and/or educators to be associated with a later sexual debut [9,21]. This may be the result of two sources of influence, including both the direct effects of education on a student as well as the indirect effects of education on a student’s peers [8]. Studies have shown that peers can be sources of both positive and negative influences on sexual behaviors [10]. In this study, nearly one third of respondents reported that some or many of their friends were sexually active, when in fact only one fifth of respondents reported having had sexual intercourse at least once. This may simply reflect the fact that peers with similar behaviors are friends and therefore are in fact more sexually active than respondents in the sample as a whole, or it indicates a general misperception among adolescents of the sexual behaviors of their peers. While the 95% confidence intervals reported in the multivariate analysis of factors associated with sexual behavior were too wide to support a conclusive finding of a significant association between perceived peer sexual experience and a respondent’s own behavior, they do suggest that peers may exert an important influence on each other that should be explored more fully. More research is needed in this population to have an accurate understanding of this relationship. The percentage of those students who consumed alcohol in this study (10%) was low in comparison to a 2013 study of students in 17 secondary schools in Botswana that found that 19.2% of students consumed alcohol [31]. There are two likely explanations for this observed difference. The earlier study included students who were attending Forms 1–5 (the US equivalent of grades 8–12) and therefore included more older students than the present study in which students were mostly enrolled in Form 2 and aged 15 years. Older students may be more likely to consume alcohol than their younger peers. The earlier study also found that alcohol consumption was higher among students in urban areas than in peri-urban settings; this would suggest that a lower alcohol consumption rate among students in Maun, a town far from a large urban center, is not surprising.

Interestingly, students who agreed with the statement ‘it is all right for boys and girls to have sex if they use a condom’ had four times greater odds of having had sexual intercourse than their peers who disagreed or were uncertain about their opinions. This may be a consequence of HIV-prevention messages targeting youth that have increasingly emphasized ‘safe sex’ over ‘no sex’ in response to limited evidence that calls for abstinence in this setting were efficacious [32]. Although not the intention of these health promotion efforts, adolescents may interpret these messages as an implicit condoning of adolescent sexual activity provided condoms are used, rather than a call for caution. 

Particularly relevant to research on adolescents in Sub-Saharan Africa and condom use is previous research in which self-efficacy, secondary education or higher, the perception that one can talk about condoms with partners, subjective norms, and behavior change prompted by HIV/AIDS were found to be positively associated with consistent condom use among adolescents in Sub-Saharan Africa [9,33,34,35,36]. Confidence in knowing how to use a condom and confidence in insisting on its use are common dimensions of condom self-efficacy. Understanding the relationship between confidence in condom use and being able to insist on condom use were shown to be significantly correlated; adolescents who reported that they did not know how to use a condom correctly had more than 70% lower odds of feeling that they could insist on condom use every time they had sex compared to those adolescents who expressed confidence regarding correct usage. 

One attitudinal statement about relationships were significant at the *p* < 0.05 level in the multivariate model examining condom use: ‘men need sex more frequently than women do‘. This statement may reflect deeply entrenched norms around gender relations that have been linked to HIV risk in this setting [37,38,39]. As such, it is indicative of the gender imbalance that pervades male–female relations and underpins a social perception that women have no power in relationships. Approximately one third of male and female adolescents agreed ‘men need sex more than women do‘. When controlling for the effects of other variables, adolescents who agreed with the statement had more than four and a half times greater odds of being confident about insisting on sex than those who disagreed. Adolescent male behavior may well be shaped by the perception that males’ greater need for sex puts them in control of all sexual negotiations. It is less clear how to interpret this finding in the context of adolescent girls, although this finding may be due to norms around what is deemed appropriate behavior for girls. Girls who carry condoms or know how to use them are often viewed in Botswana as being ‘easy’; the same may be true for how they might be viewed were they to insist on condom use.

### Limitations

There are several limitations associated with this study and its findings. Study participants were members of newly formed school clubs intended to promote healthy, equitable relationships and positive sexual and reproductive health. The extent to which these members are representative of the broader student body and, more generally, adolescents in northwestern Botswana is not clear; given their interest in an SRH club, it is possible that their responses reflect SRH attitudes and behaviors that are ‘healthier’ than those of their nonmember peers. In addition, student responses to survey questions, particularly those about behaviors that adolescents may perceive to be socially unacceptable or at odds with the behaviors of their peers, could have been influenced by the school settings in which the surveys were administered and the proximity of peers and adult authority figures. Concerns about the length of the survey dictated a parsimonious selection of variables and may have limited our ability to control for other factors that influence adolescent SRH knowledge and behaviors in this setting, including household dynamics, cultural norms among particular tribal groups, and socioeconomic status. The small sample size resulted in insufficient variability or large confidence intervals for some responses that limited meaningful interpretation of them in the multivariate analyses. Future studies would benefit from larger sample sizes that allowed for gender-stratified analyses to explore whether covariates exert the same influence on male and female behaviors. As with similar studies of adolescent HIV-risk behaviors, this study reflects attitudes and behaviors reported by the respondents themselves and therefore may reflect an under- or over-reporting of certain phenomena, including the outcome variables of interest in this study. That being said, adolescent perspectives on what constitute desirable behaviors, particularly as reflected in their perception of what peers may do or think, can help identify any misperceptions that must first be deconstructed before new ways of thinking about sexual relations, risk, and gender can be adopted.

## 5. Conclusions

Adolescent sexual behavior and condom use in northwestern Botswana are shaped by a complex web of individual, social, and structural factors that give rise to increased risk of HIV infection, unwanted pregnancies, and sexually transmitted disease. Interventions to reduce these risk behaviors and encourage good sexual and reproductive health will be successful only to the extent that they take into account the influences exerted by lack of information and the sources of that information, perceived peer behavior, norms, and attitudes relating to gender relations. Attention should be paid in school CSE curricula and other school-based initiatives in this setting to capitalize on the important role teachers play as trusted sources of information and to counter adolescents’ acceptance of normative statements that reinforce gender inequality and gender roles. Sexual education programming in Botswana is particularly important at the start of secondary school, when the majority of the students have yet to become sexually active, in order to encourage consistent condom use to prevent HIV transmission. Research involving a larger number of adolescents from the population in general would be particularly useful in generating a more in-depth understanding of sexual attitudes and behaviors among Botswana youth.

## Figures and Tables

**Table 1:** Distribution of respondents by sociodemographic attributes and behaviors according to sexual experience of the respondent (N = 233).

Variable	Values			Sexual Experience			
		*N*	%	% No	% Yes	χ^2^	*p*
Sexual experience	Yes	48	20.6				
	No	184	79.0				
	Missing	1	0.4				
Gender	Male	106	45.5	68	32	15.98	<0.001
	Female	127	54.5	89	11		
Age (years)	14	65	27.9	91	9	13.08	0.001
	15	123	52.8	79	21		
	16 and over	38	16.3	61	39		
	Missing	7	3.0				
Head of household	Father	121	51.9	82	18	5.20 ^†^	0.26
	Mother	52	22.3	76	24		
	Other male	29	12.5	79	21		
	Other female	21	9.0	71	29		
	Self	3	1.3	33	67		
	Missing	7	3.0				
Cigarette use	No	228	89.3	81	19	4.14^†^	0.103
	Yes	3	1.3	33	67		
	Missing	2	0.9				
Alcohol use	Never	208	89.3	83	17	14.53	<0.001
	Yes	24	10.3	50	50		
	Missing	1	0.4				
Drug use	Never	225	96.6	81	19	14.89 ^†^	0.001
	Yes	8	3.4	2	6		
	Missing	0	0.0				
Information source: How bodies change	Family member	112	48.1	81	19	16.89	0.001
	Teacher	72	30.9	88	12		
	Nurse/doctor	17	7.3	76	24		
	Peer	27	11.6	50	50		
	Missing	5	2.15				
Information source: Reproductive system	Family member	66	28.3	73	27	12.52	0.006
	Teacher	115	49.4	87	13		
	Nurse/doctor	19	8.2	84	16		
	Peer	27	11.6	59	41		
	Missing	6	2.6				
Information source: Relationships	Family member	78	33.5	79	21	2.99	0.393
	Teacher	68	29.2	85	15		
	Nurse/doctor	12	5.2	75	25		
	Peer	69	29.6	74	26		
	Missing	6	2.6				
Has had an HIV test	No	132	56.7	80	20	0.07	0.799
	Yes	92	39.5	81	19		
	Missing	9	3.9				
Aware of HIV status	No	124	53.2	80	20	0.00	0.966
	Yes	81	34.8				
	Missing	28	12.0				

^†^ Fisher’s exact. Missing observations were excluded in calculation of chi-square and Fisher’s exact test statistics.

**Table 2:** Distribution of respondents by sociodemographic attributes and behaviors according to gender of respondent (N = 233).

Variable	Values			Gender			
		*n*	%	% Male	% Fem	χ^2^	*p*
Sexual experience	Yes	48	20.6	71	29	16.0	<0.001
	No	184	79.0	39	61		
	Missing	1	0.4				
Gender	Male	106	45.5				
	Female	127	54.5				
Age (years)	14	65	27.9	29	71	30.74	<0.001
	15	123	52.8	41	59		
	16 and over	38	16.3	84	16		
	Missing	7	3.0				
Head of household	Father	121	51.9	47	53	4.33 ^†^	0.421
	Mother	52	22.3	40	57		
	Other male	29	12.5	45	55		
	Other female	21	9.0	43	57		
	Self	3	1.3	100	0		
	Missing	7	3.0				
Cigarette use	No	228	89.3	45	55	0.56 ^†^	0.592
	Yes	3	1.3	67	33		
	Missing	2	0.9				
Alcohol use	Never	208	89.3	45	55	0.24	0.622
	Yes	24	10.3	50	50		
	Missing	1	0.4				
Drug use	Never	225	96.6	44	56	9.93^†^	0.002
	Yes	8	3.4	100	0		
	Missing	0	0.0				
Information source: How bodies change	Family member	112	48.1	35	65	18.4	<0.001
	Teacher	72	30.9	44	56		
	Nurse/doctor	17	7.3	71	29		
	Peer	27	11.6	74	26		
	Missing	5	2.15				
Information source: Reproductive system	Family member	66	28.3	44	56	4.77	0.189
	Teacher	115	49.4	41	59		
	Nurse/doctor	19	8.2	53	47		
	Peer	27	11.6	63	37		
	Missing	6	2.6				
Information source: Relationships	Family member	78	33.5	42	58	0.76	0.86
	Teacher	68	29.2	49	51		
	Nurse/doctor	12	5.2	50	50		
	Peer	69	29.6	43	57		
	Missing	6	2.6				
Has had an HIV test	No	132	56.7	42	58	0.64	0.424
	Yes	92	39.5	48	52		
	Missing	9	3.9				
Aware of HIV status	No	124	53.2	43	57	0.00	0.947
	Yes	81	34.8	43	57		
	Missing	28	12.0				

^†^ Fisher’s exact. Missing observations were excluded in calculation of chi-Square and Fisher’s exact test statistics.

**Table 3:** Knowledge and attitudes toward condom use and relationship norms according to sexual experience of respondent (N = 233).

				Sexual Experience			
Variable	Values	*N*	%	% No	% Yes	χ^2^	*p*
Condoms are an effective way to prevent pregnancy.						0.28	0.871
	Agree	170	73.0	79	21		
	Disagree	38	16.1	79	21		
	I do not know.	24	10.3	83	17		
	Missing	2	0.4				
Condoms are an effective way to protect against HIV/AIDS.							
	Agree	159	68.2	81	19	4.27	0.119
	Disagree	33	14.2	67	33		
	I do not know.	34	14.6	85	15		
	Missing	7	3.0				
Condoms are an effective way to protect against sexually transmitted diseases.							
	Agree	145	62.2	79	21	4.50	0.105
	Disagree	31	13.3	68	32		
	I do not know.	48	20.6	88	12		
	Missing	9	3.9				
It is all right for boys and girls to have sex provided they use condoms.							
	Agree	107	45.9	71	29	8.6	0.013
	Disagree	73	31.3	88	12		
	I do not know.	46	19.7	85	15		
	Missing	7	3.0				
It would be embarrassing for someone like me to buy or obtain condoms.							
	Agree	101	43.4	85	15	3.43	0.064
	Disagree/does not know	128	54.9	75	25		
	Missing	4	1.7				
I feel I know how to use a condom correctly.							
	Agree	74	31.8	61	39	25.12	<0.001
	Disagree	71	30.5	83	17		
	I do not know.	83	35.6	93	7		
	Missing	5	2.2				
I am confident that I can insist on condom use every time I have sex.							
	Agree	124	53.2	72	28	11.80	0.003
	Disagree	33	14.2	81	19		
	I do not know	68	29.2	93	7		
	Missing	8	3.4				
I would refuse to have sex with someone not prepared to wear a condom.							
	Agree	142	60.9	79	21	4.27	0.118
	Disagree	44	18.9	70	30		
	I do not know	43	18.5	88	12		
	Missing	4	1.7				
I think that sometimes a boy has to force a girl to have sex with if he loves her.							
	Agree	29	12.5	76	24	1.84	0.398
	Disagree	169	72.5	82	18		
	I do not know	29	12.5	72	28		
	Missing	6	2.6				
It is sometimes justifiable for a boy to hit his girlfriend.							
	Agree	49	21.0	69	31	4.46	0.107
	Disagree	143	61.4	83	17		
	I do not know	32	13.7	75	25		
	Missing	9	3.9				
Men need sex more frequently than women do.							
	Agree	79	33.9	70	30	13.73	0.001
	Disagree	46	19.7	72	28		
	I do not know.	104	44.6	90	10		
	Missing	4	1.7				
Men have a right to have sex with their girlfriend whenever they wish.							
	Agree	31	13.3	45	55	25.6	<0.001
	Disagree	128	54.9	87	13		
	I do not know.	60	25.8	78	22		
	Missing	14	6.0				
If a girl has accepted cash or gifts from a man, she must have sex with him if he wants it.							
	Agree	33	14.2	67	33	4.32	0.115
	Disagree	125	53.7	83	17		
	I do not know.	68	29.2	78	22		
	Missing	7	3.0				
How many of your friends have had sexual intercourse?							
	None	73	31.3	90	10	47.21	<0.001
	Many	21	9.0	29	71		
	Some	54	23.2	68	32		
	I do not know	83	35.6	89	11		
	Missing	2	0.9				

**Table 4:** Knowledge and attitudes toward condom use and relationship norms according to gender of respondent (N = 233).

				Gender			
Variable	Values	*N*	%	% Male	% Female	χ^2^	*p*
Condoms are an effective way to prevent pregnancy.							
	Agree	170	73.0	48	52	2.46	0.292
	Disagree	38	16.1	34	66		
	I do not know.	24	10.3	46	54		
	Missing	2	0.4				
Condoms are an effective way to protect against HIV/AIDS.							
	Agree	159	68.2	47	53	5.56	0.062
	Disagree	33	14.2	58	42		
	I do not know.	34	14.6	29	71		
	Missing	7	3.0				
Condoms are an effective way to protect against sexually transmitted diseases.							
	Agree	145	62.2	44	56	0.62	0.773
	Disagree	31	13.3	52	48		
	I do not know.	48	20.6	44	56		
	Missing	9	3.9				
It is all right for boys and girls to have sex provided they use condoms.							
	Agree	107	45.9	58	42	12.73	0.002
	Disagree	73	31.3	32	68		
	I do not know.	46	19.7	41	58		
	Missing	7	3.0				
It would be embarrassing for someone like me to buy or obtain condoms.							
	Agree	101	43.4	45	44	0.05	0.816
	Disagree/does not know	128	54.9	46	54		
	Missing	4	1.7				
I feel I know how to use a condom correctly.							
	Agree	74	31.8	66	34	18.79	<0.001
	Disagree	71	30.5	37	63		
	I do not know.	83	35.6	35	65		
	Missing	5	2.2				
I am confident that I can insist on condom use every time I have sex.							
	Agree	124	53.2	52	48	6.12	0.047
	Disagree	33	14.2	45	55		
	I do not know	68	29.2	34	66		
	Missing	8	3.4				
I would refuse to have sex with someone not prepared to wear a condom.							
	Agree	142	60.9	41	59	4.54	0.103
	Disagree	44	18.9	59	41		
	I do not know	43	18.5	47	53		
	Missing	4	1.7				
I think that sometimes a boy has to force a girl to have sex with if he loves her.							
	Agree	29	12.5	62	38	5.85	0.054
	Disagree	169	72.5	40	60		
	I do not know	29	12.5	52	48		
	Missing	6	2.6				
It is sometimes justifiable for a boy to hit his girlfriend.							
	Agree	49	21.0	55	45	2.39	0.302
	Disagree	143	61.4	43	57		
	I do not know	32	13.7	41	59		
	Missing	9	3.9				
Men need sex more frequently than women do.							
	Agree	79	33.9	44	56	14.92	0.001
	Disagree	46	19.7	70	30		
	I do not know.	104	44.6	36	64		
	Missing	4	1.7				
Men have a right to have sex with their girlfriend whenever they wish.							
	Agree	31	13.3	77	23	16.15	<0.001
	Disagree	128	54.9	38	62		
	I do not know.	60	25.8	43	57		
	Missing	14	6.0				
If a girl has accepted cash or gifts from a man, she must have sex with him if he wants it.							
	Agree	33	14.2	58	42	4.02	0.134
	Disagree	125	53.7	40	60		
	I do not know.	68	29.2	50	50		
	Missing	7	3.0				
How many of your friends have had sexual intercourse?							
	None	73	31.3	37	63	13.53	0.004
	Many	21	9.0	71	29		
	Some	54	23.2	59	41		
	I do not know	83	35.6	39	61		
	Missing	2	0.9				

**Table 5:** Characteristics and behaviors associated with adolescent sexual experience and perceived ability to insist on condom use ^1^.

Variable	Sexual Experience *N* = 192			Able to Insist on Condom Use, *N* = 196		
	OR	*p*	95% CI	OR	*p*	95% CI
Gender (*ref.* male)	0.91	0.873	0.29, 2.86	0.82	0.642	0.36, 1.89
Age (*ref.* 14 years old)						
15 years old	3.57	0.096	0.80, 15.96	2.17	0.065	0.95, 4.94
16 years and over	5.30	0.054	0.97, 28.9	1.03	0.968	0.29, 3.67
Has had sexual intercourse	-	-	-	1.15	0.808	0.38, 3.45
Source of information about reproductive system (*ref.* family member)						
Teacher	0.16	0.010	0.04, 0.65	-	-	-
Nurse/doctor	0.13	0.052	0.02, 1.02	-	-	-
Peer	0.56	0.532	0.09, 3.46	-	-	-
Source of information about boys’ and girls’ bodies (*ref.* family member)						
Teacher	1.41	0.623	0.36, 5.55	1.53	0.300	0.69, 3.41
Nurse/doctor	0.62	0.637	0.08, 4.61	0.59	0.517	0.12, 2.92
Peer	2.52	0.269	0.49, 13.0	0.53	0.315	0.15, 1.83
I know how to use a condom correctly. (*ref.* agree)						
Disagree	0.99	0.987	0.26, 3.74	0.28	0.017	0.10, 0.80
I do not know.	0.38	0.186	0.09, 1.60	0.17	0.001	0.06, 0.48
I can insist on condom use every time I have sex. *(ref.* agree)						
Disagree	1.37	0.686	0.30, 6.30	-	-	-
I do not know.	0.56	0.471	0.11, 2.75	-	-	-
Men need sex more frequently than women do. (*ref.* disagree)						
Agree	1.29	0.704	0.35, 4.74	3.19	0.038	1.07, 9.55
I do not know.	0.45	0.235	0.12, 1.68	1.10	0.854	0.41, 2.91
Alcohol use (ref. no)	0.17	0.029	0.03, 0.83	-	-	-
Drug use (*ref.* no)	0.19	0.201	0.15, 2.41	1.23	0.843	0.16, 9.65
It is all right for boys and girls to have sex if they use a condom. (*ref.* disagree)						
Agree	4.12	0.038	1.08, 15.72	2.10	0.084	0.91, 4.85
I do not know.	2.46	0.282	0.48, 12.73	0.37	0.057	0.14, 1.03
How many of your friends have had sexual intercourse? (*ref.* none)						
Many	17.62	0.003	2.74, 113.52	2.80	0.213	0.55, 14.19
Some	2.23	0.273	0.53, 9.42	2.20	0.141	0.77, 6.26
I do not know.	1.27	0.752	0.29, 5.44	1.60	0.270	0.70, 3.67
McFadden’s Pseudo R-Square	0.41			0.27		

^1^ Includes only those observations for which there were responses for all variables in model.

## Data Availability

The data presented in this study are available on request from the corresponding author and may be shared under a data-use agreement with Rutgers University and WoMen Against Rape.
